# Epidemiology and Hospitalization Cost of Bladder Cancer in Kerman Province, Southeastern Iran

**Published:** 2018-04

**Authors:** Hamideh RASHIDIAN, Kazem ZENDEHDEL, Rajabali DAROUDI, Mohammad Reza EBADZADEH, Ali Akbar HAGHDOOST

**Affiliations:** 1. Dept. of Biostatistics and Epidemiology, School of Public Health, Neuroscience Research Center, Institute of Neuropharmacology, Kerman University of Medical Sciences, Kerman, Iran; 2. Cancer Research Center, Cancer Institute of Iran, Tehran University of Medical Sciences, Tehran, Iran; 3. Cancer Biology Research Center, Cancer Institute of Iran, Tehran University of Medical Sciences, Tehran, Iran; 4. Dept. of Health Economics and Management, School of Public Health, Tehran University of Medical Sciences, Tehran, Iran; 5. Dept. of Urology, Shahid Bahonar Hospital, Kerman University of Medical Sciences, Kerman, Iran; 6. Regional Knowledge Hub, and WHO Collaborating Centre for HIV Surveillance, Institute for Futures Studies in Health, Kerman University of Medical Sciences, Kerman, Iran

**Keywords:** Bladder cancer, Hospitalization cost, Iran

## Abstract

**Background:**

Bladder cancer is the fifth most common cancer in Iran. In this study, we aimed to assess the epidemiological status and calculate the hospitalization cost of bladder cancer patients in the southeastern part of Iran.

**Methods:**

This retrospective study reviewed the medical records of 243 patients admitted to a referral center for the treatment of bladder cancer patients in the southeastern part of Iran during the years 2014–2015 and extracted their pathologic and hospitalization cost data. Using Kruskal-Wallis and Mann-Witney tests, we investigated the association between hospitalization cost and other variables including sex, age, cancer grade, cancer histology, type of treatment and time from diagnosis.

**Results:**

About 53% of patients were in grade III or IV. More than half of them were non-muscle invasive (65%). The mean and median hospitalization costs per month were US$101 and US$72, respectively. The annual hospitalization cost for the first, second, and third year after diagnosis was estimated US$1608, US$840, and US$468 respectively. About 70% of patients were hospitalized only during the first year after diagnosis. In muscle-invasive bladder cancer, patients the average monthly hospitalization cost were about 2.1 times more than for non-muscle invasive patients (US$156 vs. US$76).

**Conclusion:**

Bladder cancer is a costly disease and its cost significantly varies with disease stage at diagnosis. Developing effective strategies for early detection of bladder cancer as well as careful surveillance programs for early diagnosis of recurrence could reduce the cost of this cancer.

## Introduction

Bladder cancer is one of the most common cancer diagnoses in the world, with an estimated 468351 new cases in 2015. The age standardized incidence rate (ASR) of this cancer is nine per 100000 worldwide. The ASR varies between about two per 100000 in western Africa and 12 per 100000 in southern Europe. Although the lowest incidence rates of bladder cancer are observed in Asian countries (Japan, Korea), in western Asia the incidence rate is relatively high (about 10.5 per 100000). With an ASR of 8.3 per 100000, Iran is among the ten countries with highest incidence rate in Asia (1, 2). Bladder cancer is the fifth most common cancer in Iran and approximately 5400 new bladder cancer cases occurred in 2012 (1). Kerman Province is among the provinces with a high incidence rate with ASRs in 2009 of 26.46 and 4.35 for males and females respectively (3).

More than 90% of the bladder cancers are of the transitional cell carcinoma (TCC) type. Approximately 70% to 80% of TCC are non-muscle invasive bladder cancer (NMIBC) (4, 5). NMIBC is composed of three distinctive groups: Tis, Ta, and T1 (6). Overall, more than 50% (60%–70%) of NMIBC will recur and about 10% to 30% will progress to muscle-invasive bladder cancer (MIBC) (4, 6, 7). Due to the high recurrence and progress rates in bladder cancer, treatment is costly (5). Bladder cancer is the ninth most expensive cancer in the United States in terms of overall expenditure (5). Moreover, this cancer was the most costly cancer per patient from diagnosis to death (8). The lifetime cost per NMIBC patient was estimated as between US$96000 to US$187000 in the United States (9). The total economic burden of bladder cancer was estimated as 4.9 billion euros across the European Union in 2012. Health care costs accounted for 59% of the total cost and the annual healthcare expenditure was 6942 euro per bladder cancer prevalent case (10).

Nowadays, increasing health care costs and budget limitations is one of the main concerns of healthcare policymakers. Thus health decision makers have to choose between different treatment options considering their cost and effect for optimum resource allocation (11). Although bladder cancer is one of the most common and costly cancer, data on the epidemiology and treatment cost of this cancer, especially in low and middle-income countries, including Iran, are scarce.

In this study, we aimed to assess the epidemiological characteristics and calculate the hospitalization costs of bladder cancer patients in the southeastern part of Iran.

## Materials and Methods

This was a case series study of bladder cancer patients admitted (ICD-10 Diagnosis Cod C67) to a referral center for the treatment of bladder cancer patients in the southeastern part of Iran during the years 2014–2015. We reviewed the medical records of these patients and extracted pathological and hospitalization cost data from patient medical records.

The study was approved by the Ethics Committee of Kerman University of Medical Sciences (ethical code: IR. Icmu.Rec.1394.104).

Due to the high recurrence rate of bladder cancer, usually, the majorities of patients are hospitalized several times during the years following the diagnosis and had several bill payment records. Therefore, in this study, the follow-up period was different for each patient and some patients were prevalent cases. On the other hand, medical tariffs changed significantly in Iran during the recent years especially after the Health Sector Evolution Plan of Aug 2014 (12), so in order to calculate the total hospitalization cost of each patient, all medical bill payment records from before 2015 were updated based on new tariffs for each cost item. Then we summed all of the costs from each of the patient bill payments after modification.

Since the follow-up period was different between patients we calculated the follow-up period for each patient (in month) from the date of diagnosis to the Nov 2015, then we divided the total hospitalization cost for each patient by follow-up period and calculated the average cost per month for patients. Finally, using the average hospitalization cost per month, we estimated the annual hospitalization cost for the years following the disease diagnosis. All costs were converted to US $ using the exchange rate (US$1=Rial 30158) (13).

We described patient demographic and pathologic characteristics using proportions. We tested the normality of the cost distribution using Kolmogorov-Smirnov tests and graphs. The distribution was highly right-skewed. The association between cost as dependent variable and sex, age, cancer grade, cancer histology, type of treatment and time from diagnosis as independent variables were investigated. Investigating the association between cost and the independent variables, we used non-parametric tests such as Kruskal-Wallis and Mann-Witney tests. We performed statistical analyses using the stata13 software (Stata Corp, College Station, TX, USA).

## Results

We studied 234 bladder cancer patient medical records. Eighty-three percent of patients were male. The mean (standard deviation [SD]) age of the patients was 61.7 (11.9) yr. About 53% of patients were in grade III or IV. More than half of them were non-muscle invasive (65%). About 12% of patients received chemotherapy treatment (Table 1). The mean (SD) and median hospitalization costs per month were US$101 (US$79) and US$72, respectively.

**Table 1:** Demographic and Pathological Characteristics of the study population and their association with hospitalization cost (n=234)

***Characteristics***	***Number (%)***	***Mean cost per month (US $)^a^***	**P-*value***
**Sex**			0.97
Male	195 (83.3)	105	
Female	39 (16.7)	87	
**Age (yr)^b^**			0.23
≤55	82 (35.0)	90	
55–65	68 (29.1)	114	
66–75	51 (21.8)	109	
>75	33(14.1)	105	
**Cancer grade ^b^**			<0.001
Grade I	13 (5.6)	69	
Grade II	95 (40.6)	75	
Grade III	83 (35.5)	135	
Grade IV	41 (17.5)	137	
Unknown	2 (0.8)	210	
**Muscular invasion ^b^**			0.001
Yes	77 (33.0)	156	
No	150 (64.0)	76	
Unknown	7 (3.0)	200	
**Chemotherapy**			<0.001
Yes	27 (11.5)	271	
No	207 (88.5)	86	
**Time from diagnosis (yr)**			<0.001
≤1	85 (36.3)	134	
1–2	88(37.6)	102	
2–3	36 (15.4)	80	
3–4	25 (10.7)	69	

a Comparing cost between groups we used kruskal-wallis and mann-withney test but we presented mean hospitalization cost for each group because it is more informative

b At diagnosis

There was a significant association between hospitalization cost and all variables (*P*-value ≤0.001) except for age (*P*-value =0.23) and sex (*P*-value =0.97). The average hospitalization cost per month in grade IV was about twice as much as for grade I (US$137 vs. US$69). In muscle-invasive bladder cancer patients the average hospitalization cost per month was about 2.1 times more than for non-muscle invasive patients (US$156 vs. US$76). The average monthly hospitalization cost in patients who received chemotherapy was about 3.2 times more than for patients who did not receive chemotherapy treatment (US$271 vs. US$86). Patients diagnosed less than one year had an average monthly hospitalization cost 1.9 times more than patients diagnosed 3–4 yr previously (US$134 vs. US$69) (Table 1).

Based on average hospitalization cost per month, the annual hospitalization cost for the first, second, third and fourth year after diagnosis was estimated US$1608, US$840, US$468, and US$396 respectively. Although about 75% of patients were hospitalized more than once, most of patients were hospitalized within one year after diagnosis, and lower than 30% of patients were hospitalized during the second year onwards.

## Discussion

We calculated the hospitalization cost of 234 bladder cancer patients in Bahonar Hospital of Kerman Province in southeastern Iran and reviewed their epidemiological characteristics. About half of the patients were in grade III or IV and 65% of them were NMIBC cases. The annual hospitalization cost for the first, second, third and fourth year after diagnosis was estimated US$1608, US$840, US$468, and US$396 respectively.

Although the sex distribution of bladder cancer in Iran is similar to western countries, due to the young population the mean age of patients in Iran is relatively lower (14–16). While in western countries about 70% to 80% of patients present with ‘superficial’ NMIBC, in our study about 65% of patients were non-muscle invasive cases. In another study conducted in the Fars Province of Iran, muscular invasion was seen in 54% of patients (17). Furthermore, in our study, about 36% and 18% of patients were in grades III and IV, respectively. In a study conducted on patients diagnosed with bladder cancer between 1990 and 2004 in the US, about 41% and 7% of patients were in grades III and IV, respectively (14). Compared with western countries a higher percentage of patients in Iran are diagnosed in advanced stages.

Our results showed that the hospitalization cost varies with the disease stage at diagnosis, consistent with studies conducted in the US (18–20). MIBC diagnostic costs were approximately five and three times greater than for patients with Stage Tis-Ta and Stage T1, respectively (20). MIBC treatment costs were about three times more than for patients at Stage Tis-Ta and double the costs for patients at Stage T1. In another study, the mean cost of NMIBC for initial workup, initial disease treatment, surveillance, and treatment of recurrences, was $US2401, $US6548, $US4525, and $US6401 respectively (2005 US Dollars). The corresponding values for MIBC were $US4430, $US42035, $US9768, and $US36101 respectively (19).

Transurethral resection of bladder tumor (TURBT) is the first line treatment for NMIBC patients and accounts for a considerable proportion of their total treatment costs (21). TURBT reimbursement (including costs of anesthesia, operating room, hoteling, laboratory tests and imaging) for public and private sector hospitals in Iran is about US$400 and US$1720, respectively (12). In the US, the total cost for TURBT was estimated to be $4349; however, in some European countries, the TURBT cost was estimated between US$2000 and US$2500(21). Clinical practice guidelines recommend a second TURBT 2–6 wk after an initial TURBT for high-grade NMIBC (22–24). Thus, the cost of initial treatment in high-grade NMIBC is higher than for low grade.

Another significant part of the cost of bladder cancer treatment is attributed to intravesical therapy, including immunotherapy and chemotherapy. Clinical practice guidelines currently recommend a single dose of intravesical mitomycin C given at the same time as the first TURBT in patients with initial tumors or recurrent low-grade NMIBC (22–24). The cost of a single dose of mitomycin C (40 mg) in Iran is about US$28 and US$82 in the public and private sectors, respectively (12). The corresponding cost in the US and UK is estimated at US$219 and US$87, respectively (21). Although economic evaluation studies confirmed intravesical chemotherapy as a cost-saving intervention, its use in the US remains infrequent (9, 25).

Adjuvant intravesical immunotherapy with Bacillus Calmette-Guerin (BCG) is recommended for patients with carcinoma in situ (CIS) and high-grade Ta or T1 bladder cancer (22, 23, 26). Iranian guidelines recommend a 6-wk induction course of Bacillus Calmette–Guerin (BCG) in multifocal or recurrent low-grade Ta, high-grade Ta, T1 and CIS bladder tumors. In addition, maintenance intravesical therapy with BCG is recommended for high-grade Ta, T1 and CIS bladder tumors (24). The cost of a 6-wk induction course of full-dose BCG in Iran is about US$347 and US$672 in the public and private sectors, respectively. Intravesical immunotherapy with BCG is a cost-effective treatment and the cost per year of life saved was lower than US$5000 (27–29). The frequency of use of intravesical chemotherapy and immunotherapy in NMIBC has not been reported in Iran.

A substantial part of total bladder cancer treatment cost is attributed to the cost of recurrence treatment (9). The costs of treating recurrences accounted for 39% of the total treatment cost (19). In our study due to disease recurrence, a large percent of patients were hospitalized more than once. However, most of their hospitalizations occurred within one year after diagnosis.

About 50% of NMIBC cases will have recurrent cancer detected and about 10% will progress to develop muscle-invasive cancer, which may receive another TURBT or other surgical or systemic treatments. Most recurrences (50%–60%) occurs within one year after diagnosis (8, 30). Since the probability of recurrence in high-grade NMIBC patients is greater than for low-grade patients (6), the cost of treating recurrences in high-grade patients is higher.

Due to its high recurrence and progression rates, follow-up of bladder cancer is frequent and costly. Follow-up of bladder cancer accounted for 21% of total bladder cancer treatment costs (19). The Iranian clinical practice guidelines recommend follow-up of NMIBC patients with urine analysis, cystoscopy and urinary cytology. Patients with low-grade NMIBC should be followed at three and nine months and once a year thereafter for five years. High-grade NMIBC patients should be followed every three months for the first two years, then every six months for the next two years, and once a year thereafter. Furthermore, in high-grade tumors, imaging of the upper tract must be considered everyone to two years (24).

Based on medical services tariffs in Iran, the cost of each follow-up (including the costs of urine analysis, office-based cystoscopy, urinary cytology and urologist visits) in the public and private sectors is about US$33 and US$114, respectively (12). Thus, the surveillance cost of a low-grade NMIBC patient without any recurrence or progress would be about US$198 and US$684 based on public and private sector tariffs, respectively. Moreover, in a high-grade patient without any recurrence or progress, the surveillance cost for the first five years after diagnosis would be about US$487 and US$1595 based on public and private sector tariffs, respectively. However, these estimations are based on an office-based cystoscopy tariff. If patients receive cystoscopy in hospital and under anesthesia, the cost of cystoscopy would increase by about seven times and the cost of surveillance will significantly increase.

The diagnostic and treatment procedures in patients with muscle-invasive bladder cancer (MIBC) are more invasive and costly than for NMIBC(21). Based on the disease stage and the patient condition a combination of treatment procedures including TURBT, radical cystectomy, neoadjuvant or adjuvant chemotherapy, radiation therapy and chemotherapy may be recommended for these patients. Radical cystectomy in combination with neoadjuvant or adjuvant chemotherapy is the standard treatment for locally advanced MIBC. In addition, chemotherapy with or without radiation therapy is recommended for patients with metastatic bladder cancer (23, 24, 31).

According to the data collected in this study, the average charge for radical cystectomy in a public hospital was US$1670, which based on private-sector hospital tariffs would be about US$7210. The cost of radical cystectomy in the US and UK is estimated at US$23451 and US$8090, respectively (21). The most common chemotherapy regimens for bladder cancer include the dose-dense MVAC (methotrexate, vinblastine, doxorubicin, and cisplatin) and GC (gemcitabine and cisplatin) regimens (21, 23). Since there are considerable differences between the prices of generic and brand-name drugs, the costs of these chemotherapy regimens are largely dependent on whether generic or brand name drugs are used. However, based on the average prices of the generic drugs in Iran, the costs of the dose-dense MVAC and GC regimens per cycle would be about US$142 and US$185, respectively (32).

Although clinical guidelines recommend the use of perioperative chemotherapy (neoadjuvant or adjuvant chemotherapy) in MIBC patients and several studies have confirmed its effectiveness (33–35), the use of perioperative chemotherapy in Iran is low. In our study, only about 11.5% of patients had received chemotherapy, while muscular invasion was seen in 34% of patients. In another study conducted in Iran, just 2.5% of patients received chemotherapy and about 17% were treated with a combination therapy (36). The results of a study conducted in the US showed that the utilization of perioperative chemotherapy in MIBC patients who underwent radical cystectomy increased from 29.5% in 2006 to 39.8% in 2010 (37).

As bladder cancer is more frequent in older people (30), we expect a significant increase in the incidence, prevalence and costs of this cancer in Iran in future years due to aging of the population. According to the GLOBOCAN 2012 estimation, due to demographic change the incidence of bladder cancer will be increased from 5343 in 2012 to 8412 in 2025 in Iran (1). Since the costs of diagnosis, treatment and surveillance of bladder cancer in the early stages are significantly less than for the advanced stages, diagnosing patients at an earlier stage as well as careful surveillance and early diagnosis of recurrence could reduce treatment and surveillance costs.

This is the first study that has assessed the cost of bladder cancer in Iran. However, we only calculated the inpatient cost of bladder cancer patients, as most of the follow-up procedures are done on an outpatient basis, the costs of these procedures were not calculated in our study.

## Conclusion

Bladder cancer is a costly disease and its cost significantly varies with the disease stage at diagnosis. Since a greater percentage of patients are diagnosed in advanced stages of bladder cancer in Iran, compared with western countries, developing effective strategies for the early detection of bladder cancer as well as careful follow-up programs for early diagnosis of recurrence could reduce the cost of this cancer in Iran.

## Ethical considerations

Ethical issues (Including plagiarism, informed consent, misconduct, data fabrication and/or falsification, double publication and/or submission, redundancy, etc.) have been completely observed by the authors.
